# Human Hyaluronidase PH20 Potentiates the Antitumor Activities of Mesothelin-Specific CAR-T Cells Against Gastric Cancer

**DOI:** 10.3389/fimmu.2021.660488

**Published:** 2021-07-13

**Authors:** Ruocong Zhao, Yuanbin Cui, Yongfang Zheng, Shanglin Li, Jiang Lv, Qiting Wu, Youguo Long, Suna Wang, Yao Yao, Wei Wei, Jie Yang, Bin-Chao Wang, Zhenfeng Zhang, Hui Zeng, Yangqiu Li, Peng Li

**Affiliations:** ^1^ Department of Hematology, First Affiliated Hospital, Jinan University, Guangzhou, China; ^2^ Center for Cell Regeneration and Biotherapy, Guangzhou Institutes of Biomedicine and Health, Chinese Academy of Sciences, Guangzhou, China; ^3^ University of Chinese Academy of Sciences, Beijing, China; ^4^ Guangdong Cord Blood Bank, Guangzhou, China; ^5^ Guangdong Women and Children Hospital, Panyu, Guangzhou, China; ^6^ Guangdong Lung Cancer Institute, Guangdong General Hospital (GGH) & Guangdong Academy of Medical Sciences, Guangzhou, China; ^7^ Department of Radiology, The Second Affiliated Hospital of Guangzhou Medical University, Guangzhou, China; ^8^ Key Laboratory for Regenerative Medicine of Ministry of Education, Institute of Hematology, School of Medicine, Jinan University, Guangzhou, China; ^9^ Bioland Laboratory (Guangzhou Regenerative Medicine and Health Guangdong Laboratory), Guangzhou, China

**Keywords:** CAR-T cells, immunotherapy, hyaluronic acid, gastric cancer, tumor extracellular matrix

## Abstract

T cell infiltration into tumors is essential for successful immunotherapy against solid tumors. Herein, we found that the expression of hyaluronic acid synthases (HAS) was negatively correlated with patient survival in multiple types of solid tumors including gastric cancer. HA impeded *in vitro* anti-tumor activities of anti-mesothelin (MSLN) chimeric antigen receptor T cells (CAR-T cells) against gastric cancer cells by restricting CAR-T cell mobility *in vitro*. We then constructed a secreted form of the human hyaluronidase PH20 (termed sPH20-IgG2) by replacing the PH20 signal peptide with a tPA signal peptide and attached with IgG2 Fc fragments. We found that overexpression of sPH20-IgG2 promoted CAR-T cell transmigration through an HA-containing matrix but did not affect the cytotoxicity or cytokine secretion of the CAR-T cells. In BGC823 and MKN28 gastric cancer cell xenografts, sPH20-IgG2 promoted anti-mesothelin CAR-T cell infiltration into tumors. Furthermore, mice infused with sPH20-IgG2 overexpressing anti-MSLN CAR-T cells had smaller tumors than mice injected with anti-MSLN CAR-T cells. Thus, we demonstrated that sPH20-IgG2 can enhance the antitumor activity of CAR-T cells against solid tumors by promoting CAR-T cell infiltration.

## Introduction

Adoptive T cell therapies, including chimeric antigen receptor T cells, have produced substantial responses in patients with hematological malignancies (1, 2). However, the efficacy of CAR-T cells in solid tumor patients remains uncertain, and the microenvironment of solid tumors is accepted to be a major impediment which causes the lack of efficacy. It is believed that immunosuppressive cells and molecules in the TME inhibit the activation and proliferation of T cells (3–5). In addition, the tumor extracellular matrix (ECM) has been reported to play critical roles in tumor growth and metastasis (6, 7). Overproduction of ECM components including hyaluronic acid, an extracellular glycosaminoglycan, can result in increased interstitial fluid pressure and enhanced formation of physical barrier that protects tumor cells from being attacked by immune effector cells (8–13). HA is synthesized by hyaluronic acid synthase, and HAS1, HAS2 and HAS3 have been reported to be responsible for the production of HA in human tissues. Hyaluronidases are natural enzymes able to degrade polymeric high-molecular-weight hyaluronic acid into low-molecular-weight soluble hyaluronic acid molecules. PH20, a membrane protein that is naturally expressed by human sperm, was found to possess high hyaluronidase activity. A recombinant PH20 protein has been shown to be able to enhance the penetration of chemotherapeutic drugs and infiltration of immune cells (13–16). Since low infiltration efficiency is a limitation of T cell therapy against solid cancers, and PH20 may enhance the infiltrating capacity of CAR-T cells, therefore, the combination of PH20 with CAR-T cell therapy is of translational potential and remains to be explored.

In our previous study, we evaluated the feasibility of MSLN as a target of chimeric antigen receptor T cells for gastric cancer treatment. Our data suggested that efficient T cell infiltration into tumor tissue is a prerequisite for good CAR-T cell performance (17). We then sought to further enhance the infiltrative capacity of anti-MSLN CAR-T cells by incorporating other functional elements into the CAR-T cells. In this study, we reported that co-expression of a secreted form of PH20 was able to significantly enhance the HA-degrading capacity of anti-MSLN CAR-T cells. Moreover, sPH20-IgG2-expressing anti-MSLN CAR-T cells showed improved efficiency in infiltrating solid tumor tissue and were better able to regress gastric cancer xenografts *in vivo*. Our results suggested that the expression of sPH20-IgG2 could facilitate the anti-tumor activity of CAR-T cells against solid cancer.

## Materials and Methods

### PH20 and CAR Vector Design

The human PH20 cDNA sequence was obtained from the UniProt database (ID: P38567), synthesized by Genscript Co. Ltd (Nanjing, China), and cloned into the pWPXLd-2A-eGFP lentiviral vector through the Pme1 and Spe1 restriction enzyme cloning sites, tandemly linked with a p2A peptide sequence enabling co-expression with eGFP cassette. The second-generation anti-mesothelin CAR containing a 4-1BB costimulatory domain was previously described (18), CAR expression cassette was cloned into pWPXLd lentiviral vector through pme1 and Nde1 restriction enzyme cloning sites. The transcription is driven by EF1αpromoter.

### Lentivirus Production

Lentiviral particles were produced in HEK-293T cells *via* polyethyleneimine (Sigma-Aldrich, St. Louis, MO, USA) transfection. A pWPXLd-based lentiviral plasmid and two packaging plasmids, psPAX2 and pMD.2G, were cotransfected into HEK-293T cells in 10-cm dishes at a ratio of 3:1:4 (total amount: 24 µg). Lentivirus-containing supernatants were harvested at 24, 48 and 72 hours posttransfection and filtered through a 0.45-µm filter.

### Generation of CAR T Cells

PBMCs were isolated from the buffy coats of 50-100ml peripheral blood from healthy donors using Lymphoprep (Fresenius Kabi Norge, AS, Berg i Østfold, Norway).more than 2×10^7^ T cells were negatively selected from each aliquot of PBMCs using the MACS Pan T Cell Isolation Kit (Miltenyi Biotec, Bergish Gladbach, Germany) (CD3>90%), and were counted under microscopy with typan blue exclusion (Viability>80%), and activated using microbeads coated with anti-human CD3, anti-human CD2 and anti-human CD28 antibodies (Miltenyi Biotec) at a 1:1 bead:cell ratio for 24 hours in GT-T551H3 medium (Takara Biotechnology Dalian, China) supplemented with 10% heat-inactivated fetal bovine serum (FBS), 300 IU/ml IL-2, 10 mM HEPES, 2 mM glutamine and 1% penicillin/streptomycin. Every 1×10^6^ T cells were transduced with 5-10 ml of CAR lentiviral supernatants in the presence of 8 μg/ml polybrene (Sigma) for 5 hours with 1 ml 10% FBS-containing medium, and 2 continuous rounds of transduction were conducted. After transduction, the T cells were cultured in fresh medium containing IL-2 (300 IU/mL). Subsequently, fresh medium was added every 2–3 days to maintain the cell density within the range of 0.5–1×10^6^/mL. The healthy PBMC donors who provided the primary specimens gave informed consent for the use of their samples for research purposes, and all procedures were approved by the Research Ethics Board of Guangzhou Institutes of Biomedicine and Health (GIBH).

### Cells and Culture Conditions

HEK-293T cells were maintained in Dulbecco’s modified Eagle’s medium (Gibco, Grand Island, NY, USA). BGC823 (human gastric adenocarcinoma), KATOIII (human gastric carcinoma), MKN28 (human gastric carcinoma), and MKN45 cell lines were obtained from bnbio (Beijing, China) and maintained in RPMI-1640 medium. Luciferase/GFP-expressing cell lines (BGC-823-GL and KATO-III-GL) were generated by transfection of the parental cell line with lentiviral supernatant containing luciferase-2A-GFP and were sorted for GFP expression on a FACS Aria™ cell sorter (BD Biosciences, San Jose, CA, USA). All cells were cultured at 37°C in an atmosphere of 5% carbon dioxide.

### Flow Cytometry

All samples were analyzed using a NovoCyteTM (ACEA Biosciences), LSR Fortessa or C6 flow cytometer (BD Biosciences), and data were analyzed using FlowJo software (FlowJo, LLC, Ashland, OR, USA). The antibodies used for flow cytometry included biotinylated human mesothelin (catalog 296-580) (Acrobiosystems, Beijing, China), streptavidin-APC, and anti-human CD3-APC (clone UCHT1) (BioLegend, San Diego, CA, USA). The transduction efficiency of MSLN CAR-T cells was determined by incubation of 2×10^5^ T cells with 1ug/ml biotinylated human mesothelin at 4°C for 30mins, then washed with PBS containing 1% FBS and incubated with streptavidin-APC for another 30 mins, followed by washing with PBS containing 1% FBS and resuspended with PBS for detection. All the other FACS-related staining procedures were performed on 4°C for 30min, and cells were then washed with PBS containing 1% FBS at 300g before cytometric analysis.

### Hyaluronidase Activity Assay

In total, 1×10^6^ total T cells or Jurkat cells were cultured in 2 ml of culture medium containing 200 µg high-molecular-weight hyaluronan (R&D, Catalog #GLR002) in a 48-well plate. Then, the supernatant was collected at different timepoints and centrifuged at 300 RCF for 5 min. One hundred microliters of supernatant was added to 200 µl of a solution containing 0.1% bovine serum in 24 mM anhydrous sodium acetate and 79 mM glacial acetic acid (pH 3.75). The turbidity was then recorded at 600 nm with a microplate reader.

### Tumor Killing Assays

BGC823GL target cells were incubated with Mock-T cells, anti-MSLN-T cells or anti-MSLN-sP T cells at the indicated ratio in triplicate wells of white 96-well plates. Target cell viability was monitored 18 hours later by adding 100 µl/well D-luciferin (potassium salt) (Cayman Chemical, USA) at 150 µg/ml. Background luminescence was negligible (<1% of the signal from wells containing only target cells). The percent viability (%) was calculated as the experimental signal/maximal signal×100, and the percent lysis was equal to 100% - percent viability.

### Cytokine Release Assays

ELISA kits for IL-2, IFN-γ, granzyme B and TNFa were purchased from eBioscience (San Diego, CA, USA), and all ELISAs were performed according to the manufacturer’s protocols. T cells were cocultured with target cells at an E:T ratio of 1:1 for 18 hours. The culture supernatants were then collected and centrifuged at 300 RCF for 5 min, and the supernatants were subjected to the protocols provided by the manufacturer.

### Transwell Assays

For T cell migration assays, 500 µl of GT-T551H3 medium containing 10% FBS was added into the lower chamber of an 8-µm-pore 24-well transwell plate (Corning, New York, USA). High-molecular-weight hyaluronic acid was dissolved in GT-T551H3 medium at multiple concentrations (1 mg/ml, 2 mg/ml and 5 mg/ml) and then added into the upper chamber (500 µl) to form a hyaluronic acid matrix; GT-T551H3 medium served as the negative control. Bead-activated T cells (1×10^6^) were then seeded on the top of the hyaluronic acid matrix, and at each timepoint (6 h, 12 h and 24 h), the T cells that migrated through the HA matrix into the lower chamber were counted under an optical microscope.

For Transwell killing assays, 2×10^5^ target cells (BGC823GL or KATOIIIGL) were seeded in the lower chamber of an 8-µm-pore 24-well Transwell plate in GT-T551H3 medium containing 10% FBS. Then, high-molecular-weight hyaluronic acid was dissolved in GT-T551H3 medium at multiple concentrations (1 mg/ml, 2 mg/ml and 5 mg/ml) and added to the upper chamber (500 µl) to form a hyaluronic acid matrix; GT-T551H3 medium served as the negative control. Mock-T cells, MSLN-T cells or MSLN-sP T cells were then seeded on top of the hyaluronic acid matrix at each indicated effector-to-target ratio (1:1, 1:2, and 1:4). After 48 hours, the cells in the lower chamber were digested with trypsin and centrifuged at 300 RCF for 5 min. Then, they were resuspended in 100 µl of fresh medium and seeded in a white 96-well plate, and 100 µl/well D-luciferin (potassium salt) at 150 µg/ml was then added to measure the viability of target cells as described for the tumor killing assays. The supernatant of the lower chamber in the Transwell system was also collected for cytokine release assays as described above.

### Animal Experiments

Animal experiments were performed in the Laboratory Animal Center of Guangzhou Institute of Biomedicine and Health (GIBH), Chinese Academic of Sciences. All animal procedures were approved by the Animal Welfare Committee of GIBH. All protocols were approved by the relevant Institutional Animal Care and Use Committee (IACUC). All mice were maintained in specific pathogen-free (SPF)-grade cages and were provided autoclaved food and water.

For the cell line-based gastric cancer s.c xenograft models, 1×10^6^ BGC-823 cells or 2×10^6^ MKN28 cells in 100 μL of PBS were injected subcutaneously into the right flank of NSI mice (6-8 weeks old). When tumor nodes were palpable, the mice were divided into 3 groups (Mock-T, anti-MSLN-T, and anti-MSLN-sP-T) and received 5×10^6^ CAR-T cells in 100 μL of PBS *via* the tail vein. Tumor volume was measured every 3 days with a caliper and calculated by the following equation: tumor volume =(length×width^2^)/2. On day 30(BGC823 model) or day39 (MKN28 model) after tumor injection, the mice were sacrificed, and tumor tissues were weighed and then fixed with 10% paraformaldehyde.

### Immunohistochemistry

Tumor tissue sections were fixed with 10% paraformaldehyde, embedded in paraffin, sectioned at a thickness of 4 μm, and stained using a standard hematoxylin and eosin technique. Paraffin sections were also immunostained with antibodies specific for human CD3 (Clone: OKT3, BioLegend, USA) overnight at 4°C, followed by secondary staining with goat anti-rabbit IgG (PV-9000) (ZSGB-BIO, Beijing, China). Images of all sections were obtained with a microscope (DMI6000B; Leica Microsystems, Wetzlar, Germany).

### HA Quantification Assay

Tumor samples were weighed and grinded, then centrifuged at 500g for 10min and suspensions were collected. ELISA kits for HA were purchased from Abmart Shanghai Co Ltd(Shanghai China), and test were performed according to the manufacturer’s protocols. HA content in each tumor sample were calculated as HA concentration×test volume/tumor weight(g).

### Patient Survival Analysis

Patient survival analysis was performed with gene expression profiling interactive analysis (GEPIA) database (http://gepia.cancer-pku.cn). The results were compared with Log-rank test with 95% Confidence Interval, Median survival was used as group cutoff with 50% high group and 50% low group.

### Statistics

Data are presented as the means ± standard errors of the means. Student’s t test was used to determine the statistical significance of differences between samples, Two-way ANOWA test was used to determine the statistical significance of differences between groups. A P value <0.05 indicated a significant difference. All statistical analyses were performed using Prism software version 7.0 (GraphPad, Inc., San Diego, CA, USA).

## Results

### The Expression of HAS Is Negatively Correlated With the Disease Prognosis of Solid Tumor Patients Including Gastric Cancer

**Figure 1:**
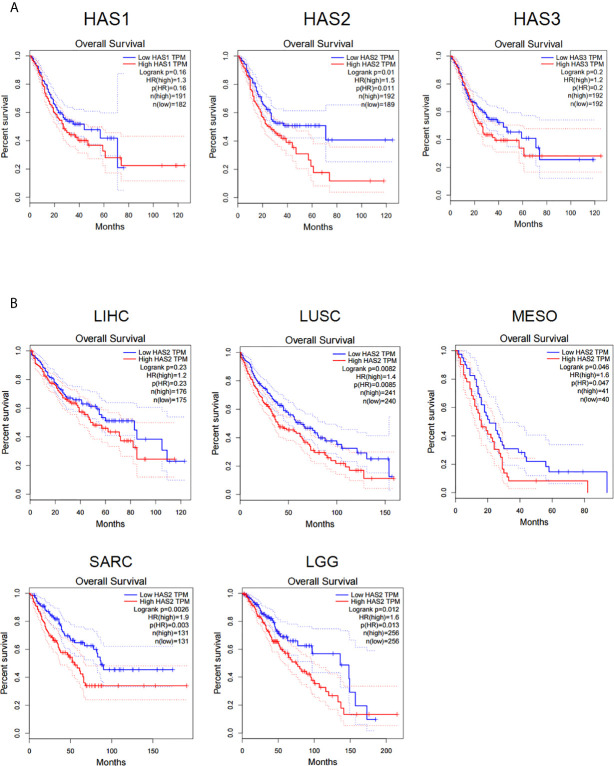
The expression of HA synthase (HAS) is negatively correlated with the disease prognosis of cancer patients. **(A)** Data analysis of the correlations of hyaluronic acid synthase expression (HAS1, HAS2, and HAS3) with patients survival in STAD patients. **(B)** Analysis for the correlations of the expression of hyaluronic acid synthase 2 (HAS2) with patient survival. LIHC, liver hepatocellular carcinoma; LUSC, lung squamous cell carcinoma; MESO, mesothelioma; SARC, sarcoma; LGG, low-grade glioma. The results were compared with Log-rank test with 95% Confidence Interval, Median survival was used as group cutoff with 50% high group and 50% low group.

We firstly analyzed the influences of hyaluronic acid synthase (HAS) expression on patient survival in the gene expression profiling interactive analysis (GEPIA) database. The results showed that the expression of all three HAS family members (HAS1, HAS2, and HAS3) was negatively correlated with patient survival in stomach adenocarcinoma (**Figure 1A**), and high HAS2 expression was negatively correlated with patient survival in multiple types of solid cancers, including hepatocellular carcinoma (LIHC), lung squamous cell carcinoma (LUSC), colon adenocarcinoma (COAD), sarcoma (SARC), and cholangiocarcinoma (CHOL) (**Figure 1B**), suggesting that a high level of HA in tumor tissues negatively impacts the prognosis of patients with solid cancers. Taken together, these results demonstrate that HAS family negatively affects patient survival in a wide range of solid cancer types, including gastric cancer.

### HA Restricts T Cell Mobility and Inhibits the Antitumor Effects of CAR-T Cells

**Figure 2:**
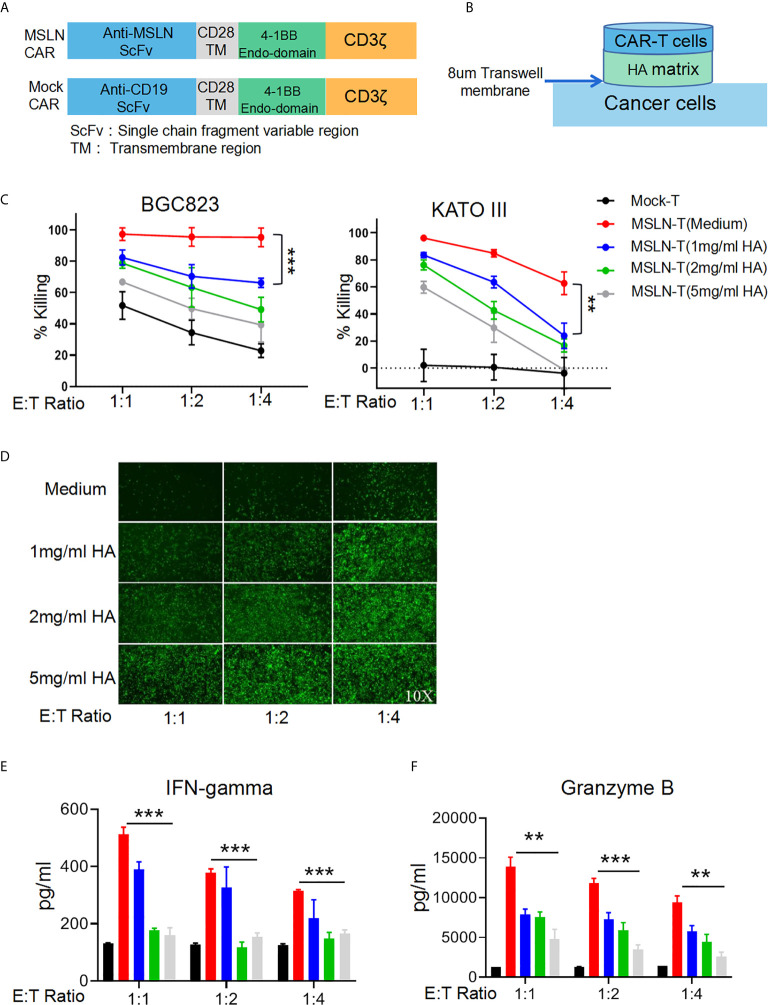
HA restricts T cell mobility and inhibits the antitumor effect of CAR-T cells. **(A)** Schematic diagram of the structures of CAR gene expression cassettes. **(B)** Schematic diagram of the Transwell tumor killing coculture system. **(C)** Results of killing assays performed with anti-MSLN CAR-T cells targeting the gastric cancer cell lines BGC823GL and KATOIIIGL in the Transwell coculture system; error bars denote the S. D, and the results were compared with two-way ANOVA. **P < 0.01. **(D)** GFP fluorescence images of viable BGC823GL cells at the endpoint of the killing assay at each E:T ratio. **(E)** Concentrations of IFN-γ and **(F)** Granzyme B in the supernatant of Transwell chambers at each E:T ratio determined by ELISA; error bars denote the S. D, and the results were compared with an unpaired t test. **P < 0.01; ***P < 0.001.

To simulate an HA-containing tumor extracellular matrix, we employed a Transwell assay to investigate the inhibitory effect of HA on T cell mobility and found that the HA concentration was negatively correlated with the number of T cells in the lower chamber at each time point (**Supplementary Figures 1A, B**), suggesting that the HA-containing matrix restricted T cell mobility. Efficient infiltration is important for the antitumor activity of CAR-T cells against solid cancers. To evaluate the inhibition of CAR-T cell mobility caused by HA, we utilized a second-generation anti-MSLN CAR containing 4-1BB as the costimulatory signal and a mock CAR with an anti-CD19 scFv (**Figure 2A**). Efficient transduction of the MSLN CAR into primary human T cells could be detected by flow cytometry with a biotinylated MSLN protein and streptavidin (**Supplementary Figure 2A**). We then seeded BGC823GL and KATOIIIGL gastric cancer cells that expressed mesothelin (17) as target cells in the lower chamber, while HA was added to the upper chamber at different concentrations, and CAR-T cells were seeded in the upper chamber in the HA matrix. Thus, the CAR-T cells that penetrated the matrix successfully migrated into the lower chamber and killed target cancer cells (**Figure 2B**). We utilized coexpression of GFP and luciferase to measure the viability of these cells with a microplate reader. The results of this assay demonstrated that HA could significantly decrease the killing activity of anti-MSLN CAR-T cells against both gastric cancer cell lines at each E:T ratio, and this effect was positively correlated with the concentration of HA (**Figure 2C**). Consistent with the luciferase assay results, the GFP intensity of BGC823GL cells in the Transwell coculture assay was much lower in the HA groups than in the control group (**Figure 2D**). Additionally, the amounts of IFN-γ and Granzyme B secreted by CAR-T cells were reduced in the HA groups compared with the medium control group (**Figures 2E, F**). Collectively, these data suggested that HA could restrict the mobility of T cells and prevent CAR-T cells from transmigrating and attacking cancer cells.

### sPH20-IgG2 Enhances the Transmigratory Capacity of Anti-MSLN CAR-T Cells in an HA-Containing Matrix and Promotes Their Anti-Tumor Activity *In Vitro*

**Figure 3:**
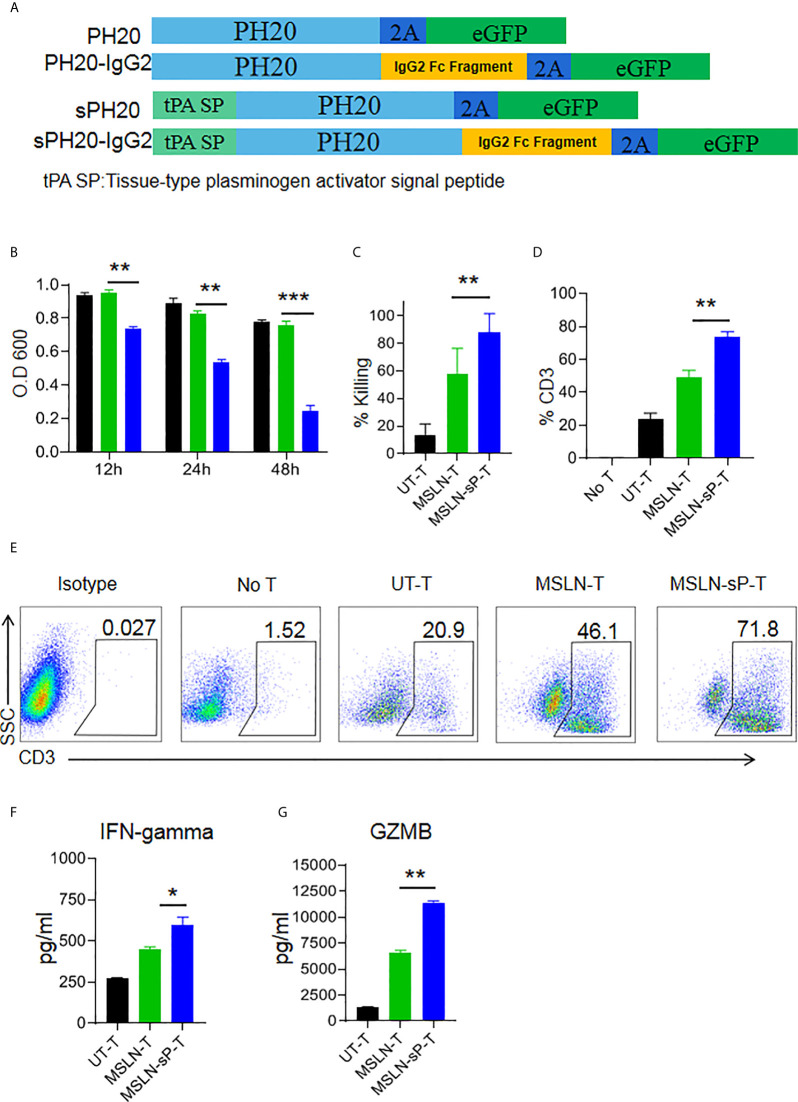
sPH20-IgG2 enhances the transmigratory capacity of anti-MSLN CAR-T cells in an HA-containing matrix and promotes their antitumor activity *in vitro.* **(A)** Schematic diagram of the four types of PH20 lentiviral expression cassettes. **(B)** Hyaluronidase activity of UT-T, MSLN-T, and MSLN-sP-T cells; error bars denote the S. D, and the results were compared with an unpaired t test. *P < 0.05; **P < 0.01; ***P < 0.001. **(C)** Viability of BGC823GL tumor cells in the lower chamber of a Transwell coculture system after coculture with UT-T, MSLN-T, or MSLN-sP-T cells at an E:T ratio of 1:1 with 5 mg/ml HA matrix in the upper chamber. **(D)** Percentage of CD3-positive T cells in the lower chamber of Transwell wells with UT-T, anti-MSLN-T, or anti-MSLN-sP-T cells at an E:T ratio=1:1 with 5 mg/ml HA matrix in the upper chamber. **(E)** Representative flow cytometry plot for **(D)**. **(F)** The concentrations of IFN-γ and **(G)** granzyme B in the supernatant of chambers in the Transwell killing assay. Error bars denote the S. D, and the results were compared with an unpaired t test. *P < 0.05; **P < 0.01; ***P < 0.001.

To test whether enforced expression of the human hyaluronidase PH20 in T cells is able to confer an enhanced capacity to degrade an HA-containing tumor extracellular matrix, we constructed four types of lentiviral expression cassettes: two membrane-located forms with or without IgG2 Fc fragments and two secreted forms with or without IgG2 Fc fragments. The secreted form of PH20 was constructed by substituting the PH20 signal peptide with the tPA signal peptide. The incorporation of IgG2 Fc fragments was intended to stabilize the structure of PH20 due to the reported short half-life of this protein (**Figure 3A**). We first transduced Jurkat cells with these gene cassettes, and the results of a hyaluronidase activity test showed that only the Jurkat cells expressing PH20-IgG2 or secreted PH20-IgG2 were able to degrade HA, with stronger activity observed in the secreted PH20-IgG2 Jurkat cells (**Supplementary Figure 3A**). The degradation of HA by secreted PH20-IgG2-expressing Jurkat cells could be detected after 24 hours compared with mock Jurkat cells (**Supplementary Figure 3B**). To test whether sPH20-IgG2 could confer hyaluronidase activity to CAR-T cells, we then cotransduced sPH20-IgG2 with the anti-MSLN CAR into primary human T cells. Coexpression of the anti-MSLN CAR and sPH20-IgG2 in T cells (anti-MSLN-sP) could be detected by FACS (**Supplementary Figure 4A**). Hyaluronidase activity measurements showed that the expression of sPH20-IgG2 enhanced the hyaluronidase activity of anti-MSLN CAR-T cells (**Figure 3B**). In vitro killing and cytokine secretion assays suggested that the expression of sPH20-IgG2 did not affect the cytotoxicity or cytokine secretion of anti-MSLN CAR-T cells (**Supplementary Figures 4B, C**). Next, we again utilized a Transwell coculture system to evaluate the antitumor activity of anti-MSLN-sP T cells in the context of an HA-containing ECM. The results showed that the anti-MSLN-sP CAR-T cells were better able to attack target cancer cells (**Figure 3C**) and penetrate through the HA-containing matrix than conventional anti-MSLN CAR-T cells (**Figures 3D, E**). The amounts of IFN-*γ* and granzyme B in the lower chamber were also increased in the anti-MSLN-sP CAR-T cell group compared with the conventional anti-MSLN CAR-T cell group (**Figures 3F, G**). Collectively, these results suggested that sPH20-IgG2 expressed by CAR-T cells was able to degrade HA and enhanced the capacity of CAR-T cells to transmigrate through the HA-containing matrix.

### sPH20-IgG2 Enhances the Infiltration and Antitumor Activity of Anti-MSLN CAR-T Cells in Xenograft Gastric Cancer Mouse Models

**Figure 4:**
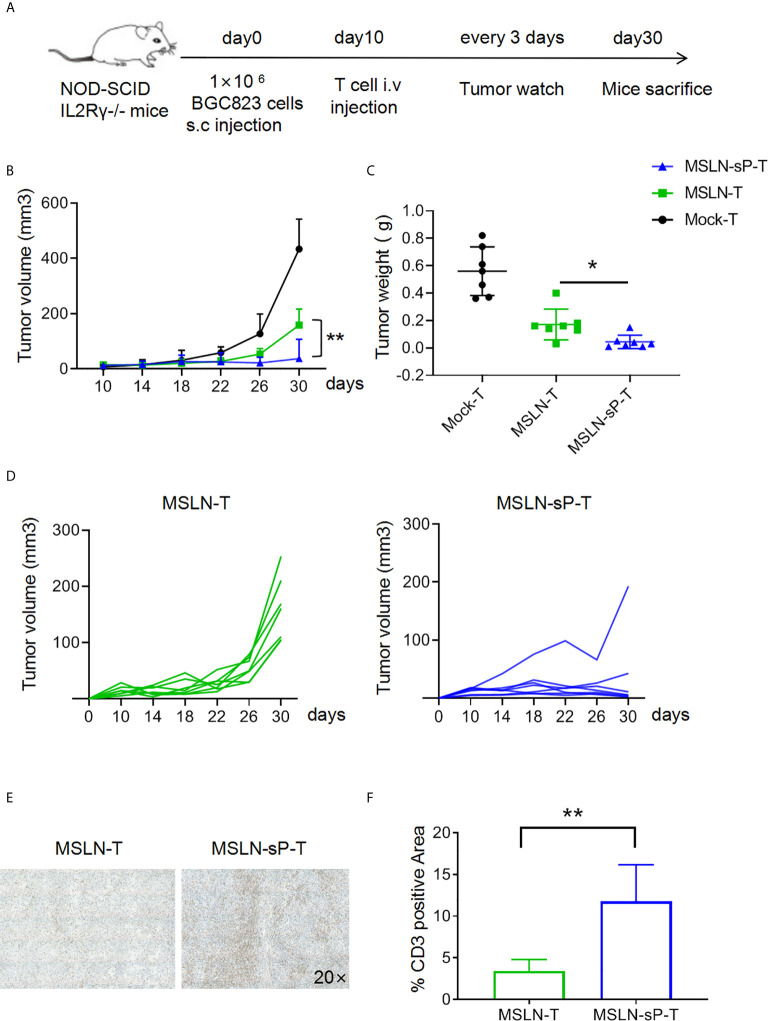
sPH20-IgG2 enhances the infiltration and antitumor activity of anti-MSLN CAR-T cells in BGC823 gastric cancer mouse model. **(A)** Schematic diagram of the experiment,n=7 for each group. **(B)** Measurement of tumor volumes after treatment with Mock-T, anti-MSLN-T or anti-MSLN-sP-T cells; error bars denote the S. D, and the results were compared with two-way ANOVA. *P < 0.05, **P < 0.01. **(C)** Tumor weights of the Mock-T, anti-MSLN-T and anti-MSLN-sP-T cell groups on day 30 after tumor injection (time of sacrifice). **(D)** Tumor growth curves for the individual mice in the anti-MSLN-T and anti-MSLN-sP-T cell groups. **(E)** Representative images of IHC detection of tumor-infiltrated T cells in the anti-MSLN-T and anti-MSLN-sP-T cell groups. **(F)** Statistical analysis of tumor-infiltrated T cells in the anti-MSLN-T and anti-MSLN-sP-T cell groups. Error bars denote the S. D, and the results were compared with an unpaired t test. *P < 0.05; **P < 0.01.

To test the functions of sPH20-IgG2-expressing CAR-T cells *in vivo*, we utilized cell line-derived xenograft mouse model. Firstly, NSI mice were subcutaneously inoculated with 1×10^6^ BGC823 human gastric cancer cells. After 10 days, when tumors were palpable, 5×10^6^ Mock-T cells, anti-MSLN CAR-T cells or anti-MSLN-sP CAR-T cells were injected *via* the tail vein. Tumor growth was monitored with a caliper. After 30 days, the mice were sacrificed, and the tumor tissues were weighed and dissected for IHC analysis (**Figure 4A**). The results showed that the conventional anti-MSLN CAR-T cells could significantly inhibit tumor growth compared with the Mock-T cells in these mice; However, the anti-MSLN-sP CAR-T cells showed a significantly stronger capacity to regress tumors, as depicted by combined tumor growth curves and tumor weight measurements (**Figures 4B, C**), as well as individual tumor growth curves (**Figure 4D**). Immunohistochemical staining for CD3 suggested that T cell infiltration in the anti-MSLN-sP CAR-T cell group was significantly higher than that in the conventional anti-MSLN CAR-T cell group (**Figures 4E, F**).

**Figure 5:**
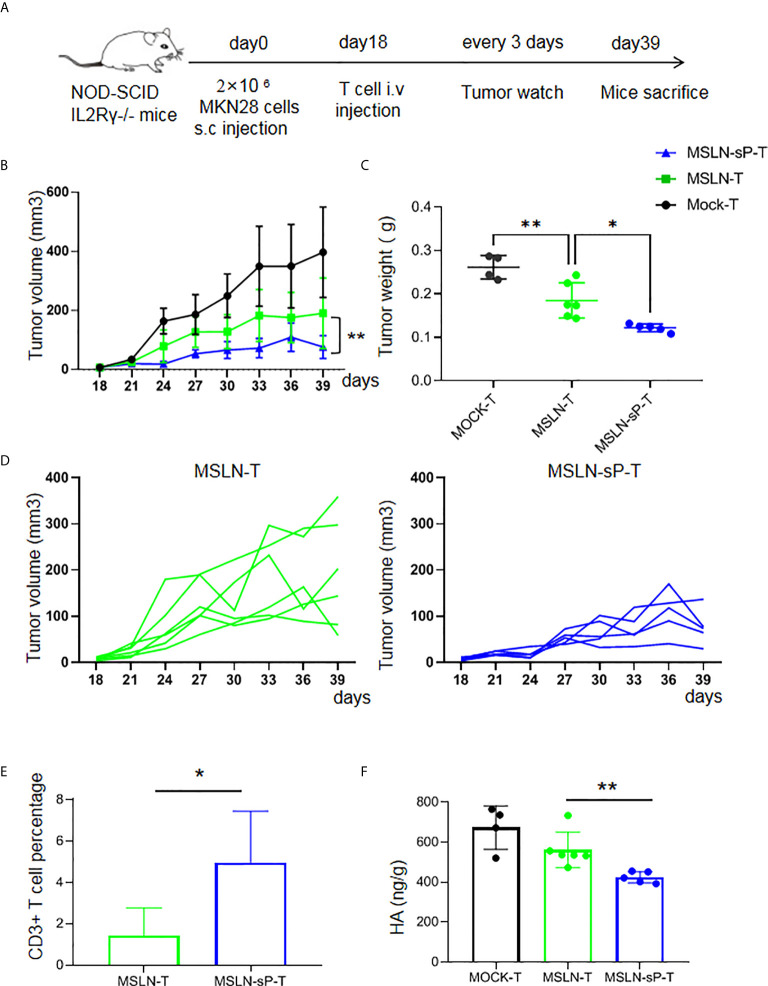
sPH20-IgG2 enhances the infiltration and antitumor activity of anti-MSLN CAR-T cells in MKN28 gastric cancer mouse model. **(A)** Schematic diagram of the experiment, n=4 for Mock-T, n=6 for MSLN-T and n=5 for MSLN-sP-T group. **(B)** Measurement of tumor volumes after treatment with Mock-T, anti-MSLN-T or anti-MSLN-sP-T cells; **(C)** Tumor weights of the Mock-T, anti-MSLN-T and anti-MSLN-sP-T cell groups on day 39 after tumor injection (time of sacrifice). **(D)** Tumor growth curves for the individual mice in the anti-MSLN-T and anti-MSLN-sP-T cell groups. **(E)** Statistical analysis for the percentage of T cells in the tumor tissues of the anti-MSLN-T and anti-MSLN-sP-T cell treated groups. **(F)** ELISA detection of HA level in the in the tumor tissues of the anti-MSLN-T and anti-MSLN-sP-T cell treated groups. Error bars denote the S. D, and the results were compared with an unpaired t test. *P < 0.05; **P < 0.01.

Despite the above result, our drawback is the limited co-expression efficiency of sPH20-IgG2 with MSLN CAR. To improve the co-expression efficiency, we flow-sorted the GFP+ CAR MSLN+ double positive T cells which represents the co-expression of sPH20-IgG2 with MSLN CAR, and expanded these sorted cells *in vitro*. The results showed that the majority of sorted cells were GFP+ CAR MSLN+ double positive cells (**Supplementary Figure 5A**), and they possess substantial hyaluronidase activity *in vitro* (**Supplementary Figure 5B**).To further verify the *in vivo* efficacy of these T cells, we utilized another gastric cancer mouse model in which 2×10^6^ MKN28 human gastric cancer cells were subcutaneously inoculated with NSI mice. After 18 days, when tumors were palpable, 5×10^6^ Mock-T cells, anti-MSLN CAR-T cells or anti-MSLN-sP CAR-T cells were injected *via* the tail vein. Tumor growth was monitored with a caliper. After 39 days, the mice were sacrificed, and the tumor tissues were weighed (**Figure 5A**). The results showed that the anti-MSLN-sP CAR-T cells again exhibited stronger capacity to regress tumors (**Figures 5B–D**). Meanwhile, T cell infiltration in the anti-MSLN-sP CAR-T cell group was significantly higher than that in the anti-MSLN CAR-T cell group (**Figure 5E**). We then detected the level of HA in the tumor tissues by ELISA assay, and found that HA level was decreased in anti-MSLN-sP CAR-T cell treated groups compared with anti-MSLN CAR-T cell group (**Figure 5F**). Taken together, these results demonstrated that the expression of sPH20-IgG2 was able to enhance the tumor infiltration of anti-MSLN CAR-T cells and potentiate their anti-tumor activity against gastric cancer *in vivo*.

## Discussion

It has been widely reported that the tumor microenvironment suppresses the antitumor activities of T cells *via* immunosuppressive cells and molecules (19, 20). Multiple strategies targeting these cells and molecules have been developed or investigated (21–24). In addition, many solid tumors produce high levels of ECM components. Collagen, proteoglycans, laminins and HA have been recognized as major ECM molecules (25–27). The physical barrier produced by the overproduction of ECM molecules in tumor tissues prevents infiltration of immune cells and protects tumor cells from immune attack (9, 28–30). Therefore, degradation of the tumor ECM is an important process for the efficient trafficking and accumulation of T cells in tumor sites (31). HA, a glycosaminoglycan that is not conjugated with a peptide, is synthesized by a family of three transmembrane glycosyltransferases, termed hyaluronan synthetase1-3 (8, 32). Of note, HAS-2 is the most commonly expressed HAS isoform according to literature and it is reported to catalyze the synthesis of high molecular weight forms of HA, which may contribute to the formation of abnormal physical barrier to prevent immune cell infiltration (9, 10). Whereas HAS-3 drives the production of large amounts of low molecular weight chains of HA (9, 11). This may be the reason for the observation in our patient survival analysis which showed that HAS2 exhibits much stronger negative correlation with patient’s survival than the other two HAS family members. HA overproduction in tumors increases tissue interstitial fluid pressure and stiffness, both of which impede T cell migration and physical contact with tumor cells (9, 10). Therefore, overcoming the physical barrier established by HA is important for improving the antitumor activity of CAR-T cells against solid tumors.

To date, how ECM molecules affect T cell therapy remains unknown. One group reported that overexpression of heparanase (HPSE) in CAR-T cells enhanced the abilities of the cells to degrade heparan sulfate proteoglycans and infiltrate tumor tissues (33). Therefore, ECM molecules are potential targets to improve the efficacy of T cell therapies. Considering the diverse composition of the ECMs of different tumors, strategies targeting different ECM molecules should be developed and may be incorporated to further improve the efficacy of CAR-T cell therapies. To target HA, some studies have applied PH20 with chemotherapies, monoclonal antibodies or oncolytic viruses. Research by Paolo and colleagues demonstrated that HA was enriched in the stroma of pancreatic ductal adenocarcinomas (PDAs) and that administration of PH20 with the conventional chemotherapeutic agent gemcitabine remodeled the tumor microenvironment and vessels, thereby improving antitumor efficacy (13). Netai et al. demonstrated that HA restricted the efficacy of monoclonal antibodies and that PEGylated PH20 could degrade the tumor pericellular matrix and enhance antibody-dependent cytotoxicity (ADCC) (16). Sonia et al. reported the construction of a PH20-overexpressing oncolytic adenovirus and showed that this modification enhanced the intratumoral spread of viruses, which resulted in better tumor control (34). Recently, Xiong et al. reported the construction of GPC3 targeted CAR-T cells with the overexpression of IL-7 and PH20 and found these two elements together can promote the tumor suppressor activity of these CAR-T cells *in vitro* and *in vivo (* 35). Collectively, these reports and our present study together suggest the potential advantages of human hyaluronidase PH20 in cancer therapies. Considering the great heterogenity of solid tumors, detailed analysis for the composition of tumor ECM will make contribution for the development of therapeutic strategies targeting ECM molecules, and the safety of these strategies need to be tested in the suitable humanized animal models, which is also the limitation of the current study.

In the present study, we found that sPH20-IgG2-expressing CAR-T cells exhibited a strong capacity to degrade the hyaluronic acid matrix *in vitro* and showed an improved ability to suppress tumor growth in a xenograft gastric cancer mouse model. Our study highlights that PH20 can serve as an attractive functional element for adoptively transferred T cells, including CAR-T cells, to potentiate their infiltration into solid tumors and that this strategy is of substantial significance and should be tested in other research models or in the clinic.

## Data Availability Statement

The original contributions presented in the study are included in the article/**Supplementary Material**. Further inquiries can be directed to the corresponding authors.

## Ethics Statement

The animal study was reviewed and approved by Animal Welfare Committee of Guangzhou Institute of Biomedicine and Health, Chinese Academy of Sciences.

## Author Contributions

RZ, YC, YZ, SL, and JL contributed to the conception and design, the collection and/or assembly of data, data analysis and interpretation, and manuscript writing. QW, YgL, and SW provided animal care and administrative support. YY, WW, JY, BW, and ZZ contributed to the conception and design of the study. YqL and PL contributed to the conception and design of the study, data analysis and interpretation, manuscript writing, and the final approval of the manuscript and provided financial support. All authors contributed to the article and approved the submitted version.

## Funding

This study was supported by Strategic Priority Research Program of the Chinese Academy of Sciences, No. XDB19030205 (PL); National Natural Science Foundation of China, No. 81961128003 (PL), 81972672 (PL), 81773301 (ZJ) 81870121 (PL), 81873847 (JY); National Key Research and Development Plan, No. 2017YFE0131600 (YL), 2019YFA0111500 (XL); The National Major Scientific and Technological Special Project for “Significant New Drugs Development”, No. 2018ZX090201002-005; the Youth Innovation Promotion Association of the Chinese Academy of Sciences (2020351, ZJ); Guangdong Provincial Significant New Drugs Development, No. 2019B020202003 (PL); Guangdong Special Support Program, No. 2017TX04R102 (PL); Guangdong Basic and Applied Basic Research Foundation, No. 2017A030310381 (ZJ), 2019A1515010062 (YY), NO. 2020A1515011516 (XW); Guangzhou Science and Technology Plan Project, No. 201907010042 (PL); 201904010473 (ZJ); Frontier Research Program of Guangzhou Regenerative Medicine and Health Guangdong Laboratory, No. 2018GZR110105003 (PL); Science and Technology Program of Guangzhou, China (202002020083, XL); Guangzhou Medical University High-level University Construction Research Startup Fund, NO. B195002004013 (LQ); Open project of State Key Laboratory of Respiratory Disease, SKLRD-OP-202002 (ZZ).

## Conflict of Interest

The authors declare that the research was conducted in the absence of any commercial or financial relationships that could be construed as a potential conflict of interest.

## Supplementary Material

Supplementary Figure 1 HA inhibits the transmigratory capacity of T cells in *vitro*. **(A)** Microscopy images of human T cells in the lower chamber of Transwell wells with different concentrations of HA (medium only, 1 mg/ml, 2 mg/ml, or 5 mg/ml) at different timepoints (6 h, 12 h, and 24 h). **(B)** Cell counts for T cells in the lower chamber of Transwell wells with different concentrations of HA (medium only, 1 mg/ml, 2 mg/ml, or 5 mg/ml). Error bars denote the S. D, and the results were compared with two-way ANOVA. *P < 0.05, **P < 0.01.

Supplementary Figure 2 Representative flow cytometry image of CAR expression efficiency in transduced T cells.

Supplementary Figure 3 In vitro functional screening of PH20-overexpressing vectors. **(A)** Hyaluronidase activity of PH20-IgG2-, PH20-, sPH20-IgG2-, and sPH20-transduced Jurkat cells, untransduced Jurkat cells and a medium control at 24 hours. **(B)** Hyaluronidase activity of sPH20-IgG2-transduced Jurkat cells, untransduced Jurkat cells and the medium control at different timepoints (12 h, 24 h and 48 h). Error bars denote the S. D, and the results were compared with an unpaired t test. *P < 0.05; **P < 0.01, ***P < 0.001.

Supplementary Figure 4 Expression of sPH20-IgG2 does not affect the normal effector functions of anti-MSLN CAR-T cells. **(A)** Flow cytometric analysis of untransduced, anti-MSLN CAR-transduced, and anti-MSLN-sP CAR-transduced T cells. **(B)** Results of a direct killing assay with Mock-T, anti-MSLN-T or anti-MSLN-sP-T cells and the target gastric cancer cell line BGC823GL. Error bars denote the S. D, and the results were compared with two-way ANOVA. **(C)** Secretion of IFN-γ and Granzyme B by Mock-T, anti-MSLN-T and anti-MSLN-sP-T cells after coculture with BGC823GL cells for 24 h. Error bars denote the S. D, and the results were compared with an unpaired t test. n.s: not significant.

Supplementary Figure 5
**(A)** Flow cytometric analysis of flow sorted anti-MSLN-sP CAR-transduced T cells.**(B)** Hyaluronidase activity of Mock-T, anti-MSLN-T and anti-MSLN-sP-T cells after flowcytometry sorting of CAR MSLN+GFP+ double positive population.Error bars denote the S. D, and the results were compared with an unpaired t test. *P < 0.05; **P < 0.01, ***P < 0.001.
