# Supplementary figures and images for: Human Hyaluronidase PH20 Potentiates the Antitumor Activities of Mesothelin-Specific CAR-T Cells Against Gastric Cancer
Source: Front Immunol. 2021 Jul 13;12:660488. doi: 10.3389/fimmu.2021.660488 (PMC8313856; doi:10.3389/fimmu.2021.660488)

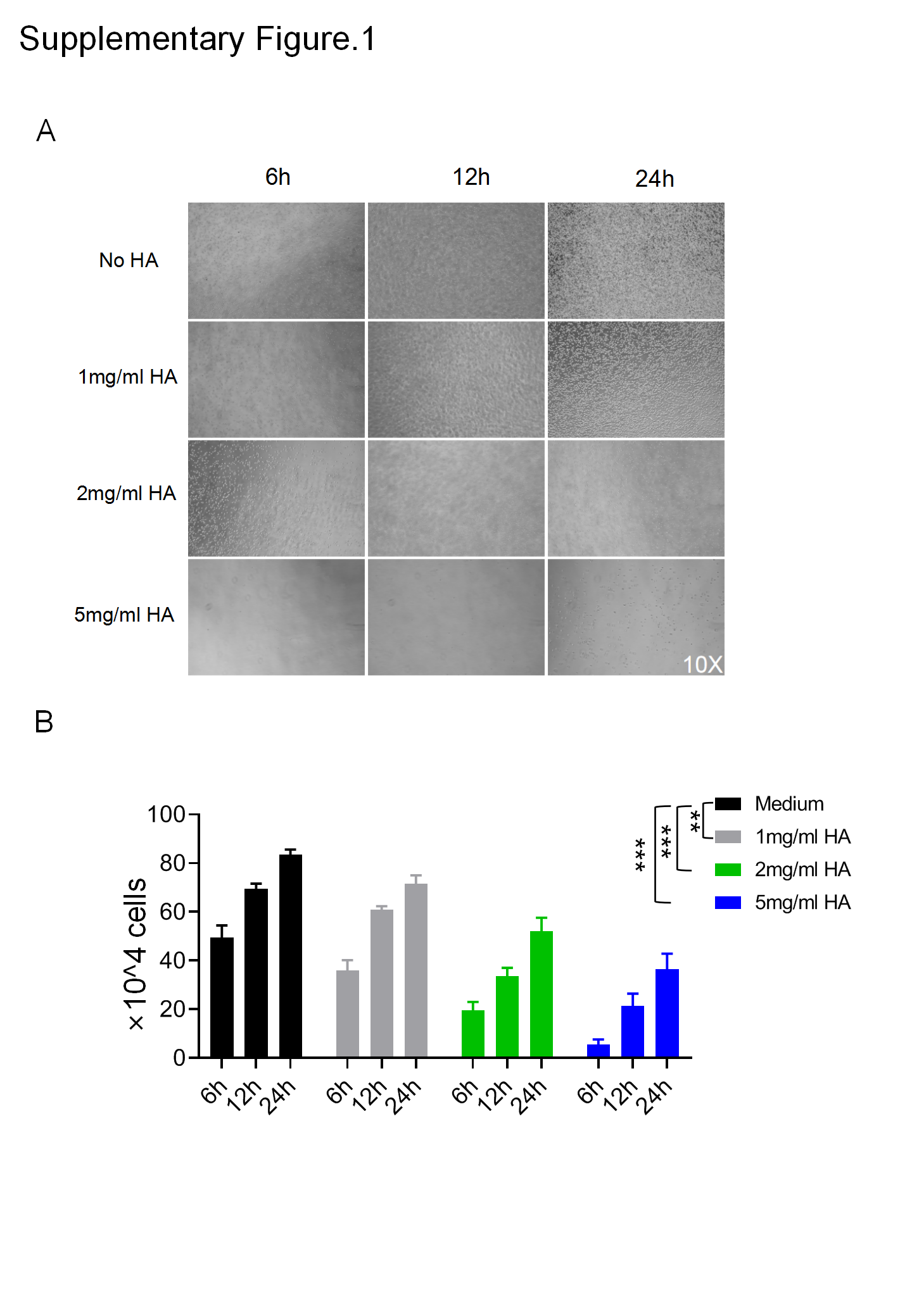

Supplement: Supplementary Figure 1 — HA inhibits the transmigratory capacity of T cells in vitro. (A) Microscopy images of human T cells in the lower chamber of Transwell wells with different concentrations of HA (medium only, 1 mg/ml, 2 mg/ml, or 5 mg/ml) at different timepoints (6 h, 12 h, and 24 h). (B) Cell counts for T cells in the lower chamber of Transwell wells with different concentrations of HA (medium only, 1 mg/ml, 2 mg/ml, or 5 mg/ml). Error bars denote the S. D, and the results were compared with two-way ANOVA. *P < 0.05, **P < 0.01. [file Image_1.tif]

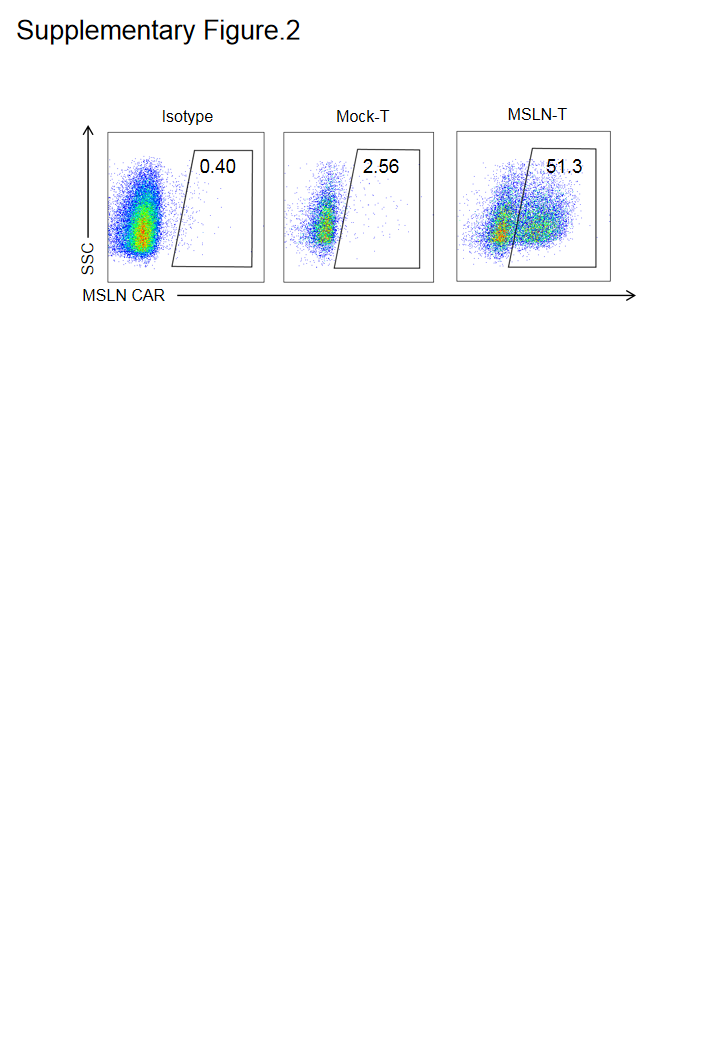

Supplement: Supplementary Figure 2 — Representative flow cytometry image of CAR expression efficiency in transduced T cells. [file Image_2.tif]

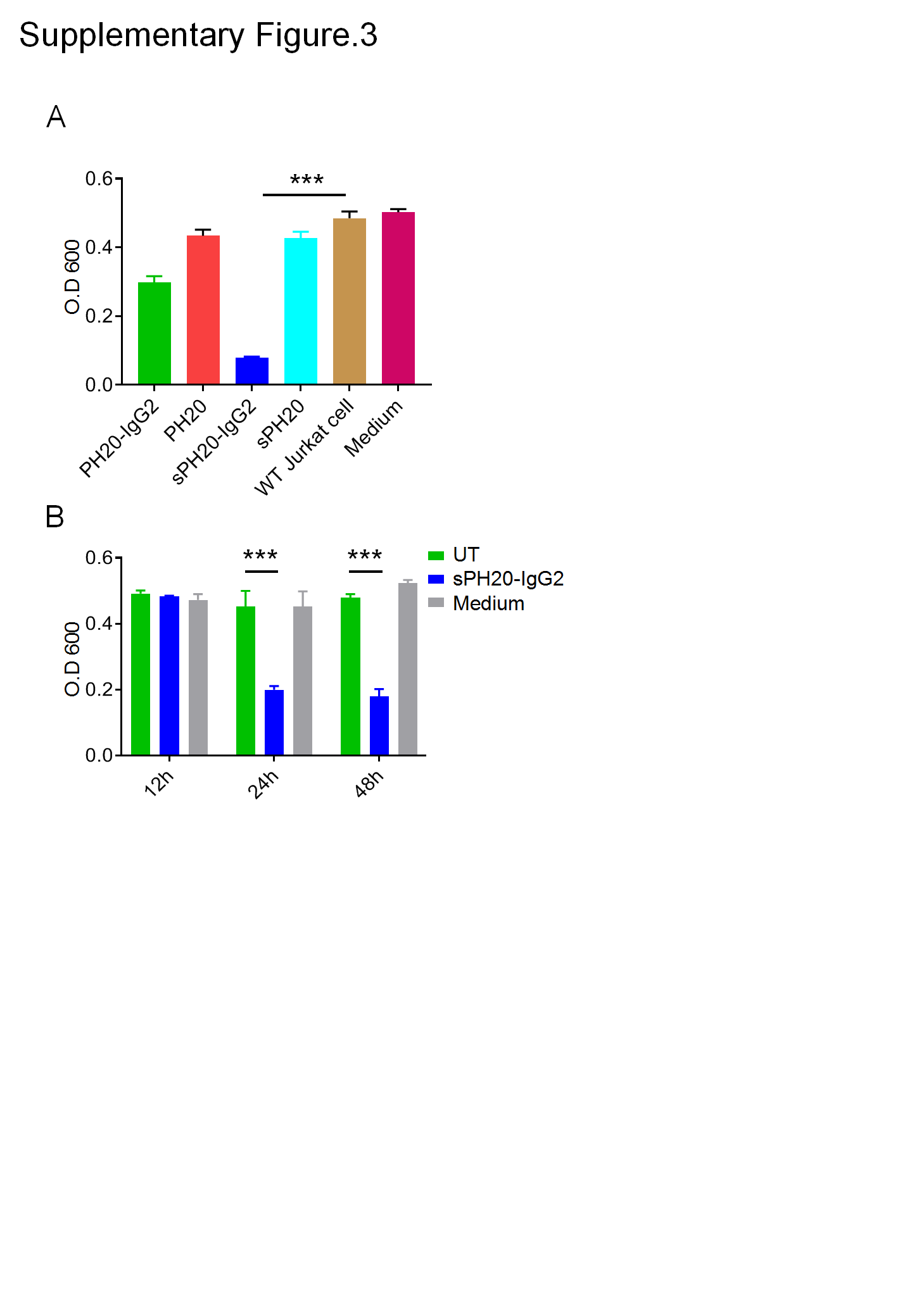

Supplement: Supplementary Figure 3 — In vitro functional screening of PH20-overexpressing vectors. (A) Hyaluronidase activity of PH20-IgG2-, PH20-, sPH20-IgG2-, and sPH20-transduced Jurkat cells, untransduced Jurkat cells and a medium control at 24 hours. (B) Hyaluronidase activity of sPH20-IgG2-transduced Jurkat cells, untransduced Jurkat cells and the medium control at different timepoints (12 h, 24 h and 48 h). Error bars denote the S. D, and the results were compared with an unpaired t test. *P < 0.05; **P < 0.01, ***P < 0.001. [file Image_3.tif]

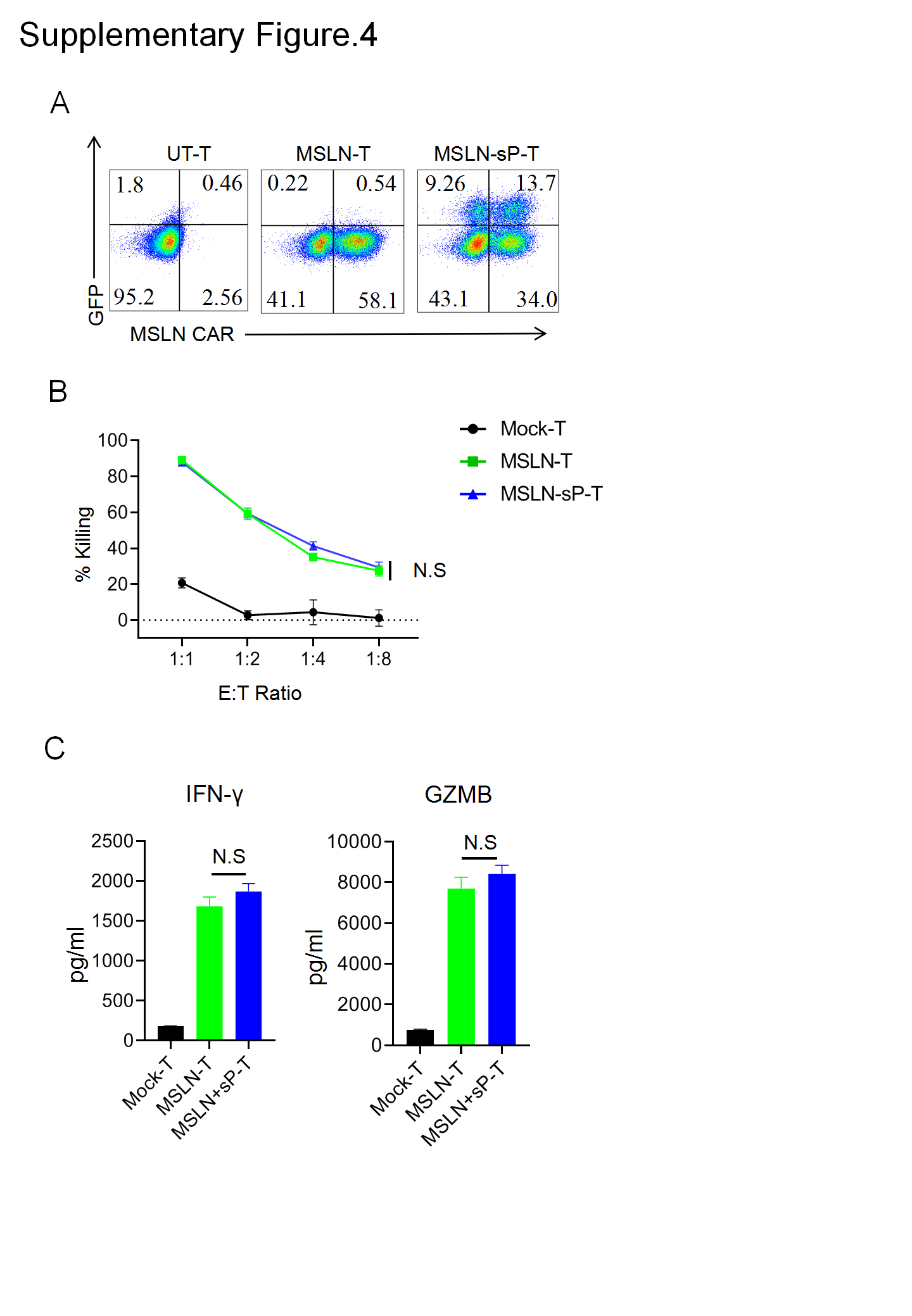

Supplement: Supplementary Figure 4 — Expression of sPH20-IgG2 does not affect the normal effector functions of anti-MSLN CAR-T cells. (A) Flow cytometric analysis of untransduced, anti-MSLN CAR-transduced, and anti-MSLN-sP CAR-transduced T cells. (B) Results of a direct killing assay with Mock-T, anti-MSLN-T or anti-MSLN-sP-T cells and the target gastric cancer cell line BGC823GL. Error bars denote the S. D, and the results were compared with two-way ANOVA. (C) Secretion of IFN-γ and Granzyme B by Mock-T, anti-MSLN-T and anti-MSLN-sP-T cells after coculture with BGC823GL cells for 24 h. Error bars denote the S. D, and the results were compared with an unpaired t test. n.s: not significant. [file Image_4.tif]

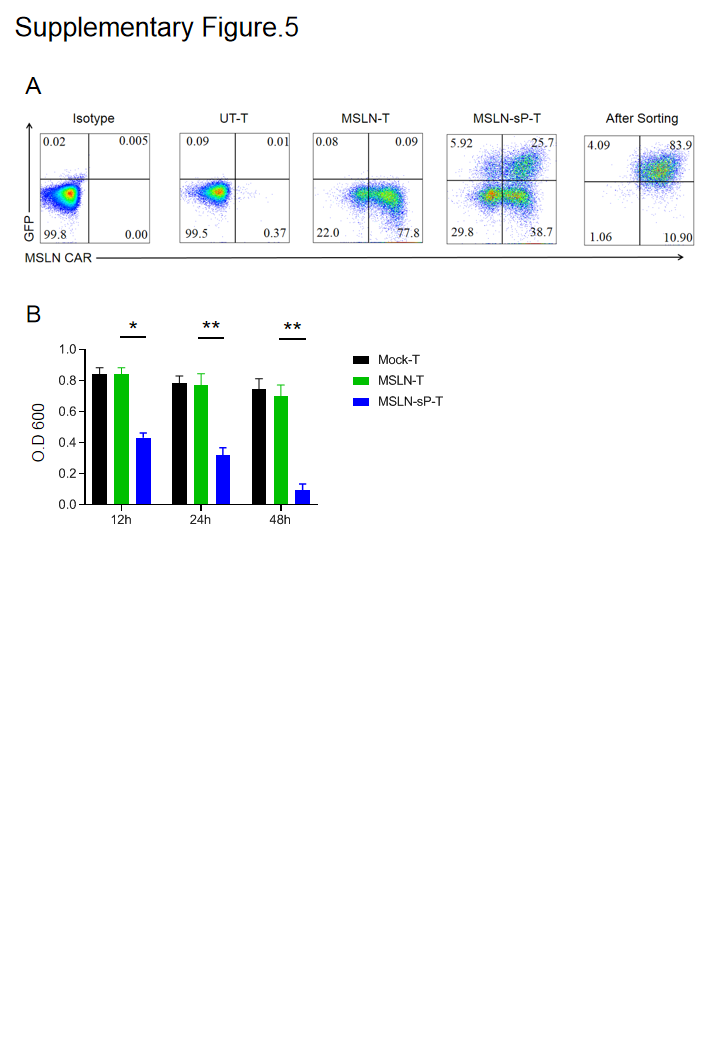

Supplement: Supplementary Figure 5 — (A) Flow cytometric analysis of flow sorted anti-MSLN-sP CAR-transduced T cells.(B) Hyaluronidase activity of Mock-T, anti-MSLN-T and anti-MSLN-sP-T cells after flowcytometry sorting of CAR MSLN+GFP+ double positive population.Error bars denote the S. D, and the results were compared with an unpaired t test. *P < 0.05; **P < 0.01, ***P < 0.001. [file Image_5.tif]
